# The effect of creep feed composition and form on pre- and post-weaning growth performance of pigs and the utilization of low-complexity nursery diets

**DOI:** 10.1093/tas/txab211

**Published:** 2021-11-10

**Authors:** Brenda Christensen, Lee-Anne Huber

**Affiliations:** Department of Animal Biosciences, University of Guelph, Guelph, ON, Canada

**Keywords:** creep feed, growth performance, milk replacer, nursery diet complexity, pigs

## Abstract

Fifty-six litters from first-parity sows standardized to 12 piglets were used to determine the effects of creep feed composition and form on pre- and post-weaning pig growth performance and the utilization of low-complexity nursery diets. At 5 days of age, litters (initial body weight [BW] 2.31 ± 0.61 kg) were assigned to one of four creep feeding regimens (*n* = 14): 1) pelleted commercial creep feed (COM), 2) liquid milk replacer (LMR), 3) pelleted milk replacer (PMR), or 4) no creep feed (NO); creep feeds contained 1.0% brilliant blue as a fecal marker. Individual piglet BW and fecal swabs were collected every 3 ± 1 days during the creep-feeding period. The latter was to identify piglets that regularly consumed creep feed via the visual appearance of blue dye in the feces. At weaning (21 ± 2 days of age), six pigs per litter with median BW that consumed creep feed were placed on either a HIGH− (contained highly digestible animal proteins) or LOW− (contained corn and soybean meal as the main protein sources) complexity nursery diet (*n* = 7) in a three-phase feeding program over 39 days. On day 8, two pigs per pen were sacrificed to collect organ weights and digesta. The LMR disappeared at the greatest rate (average 37.7 g/pig/d; dry matter-basis) versus COM and PMR (10.6 and 10.3 ± 1.5 g/pig/d, respectively; *P* < 0.001). Litters that received LMR had the greatest proportion of pigs with blue fecal swabs throughout the creep feeding period (85.0 vs. 54.9 and 63.0% ± 0.4% for COM and PMR, respectively; *P* < 0.05) and LMR piglets had greater BW at weaning versus all other treatments (6.32 vs. 6.02, 5.92, and 5.67 ± 0.14 kg, for LMR, COM, NO, and PMR, respectively; *P* < 0.001). Overall, pigs given LOW (vs. HIGH) diets in the nursery period had reduced average daily gain (25.1 vs. 27.7 ± 0.4 g/kg BW; *P* < 0.001), gain:feed (0.75 vs. 0.81 ± 0.02; *P* < 0.001), and exit BW (21.2 vs. 24.4 ± 0.6 kg; *P* < 0.001); no carryover effects of creep feeding program were observed. Creep feed regimen had limited effects on nutrient digestibility of nursery diets but the apparent ileal digestibility of organic matter tended to be less at 28 days of age for pigs that received the LOW nursery diet (64.2 vs. 68.8% ± 2.5%; *P* = 0.076). Providing supplemental nutrition during the suckling period via LMR improved piglet BW at weaning, which did not correspond to improved post-weaning growth performance, regardless of nursery diet complexity.

## INTRODUCTION

Providing supplemental feed to piglets (i.e., creep feed) during the lactation period is one strategy to increase energy and nutrient intakes of piglets, maximize piglet body weight (BW) at weaning, and potentially, reduce nutrient demands for the lactating sow (Miller et al., 2012; Novotni-Dankó et al., 2015). Creep feed consumption is highly variable between and within litters (Pajor et al., 1991; Bruininx et al., 2002), but piglets that consume creep feed have greater average daily gain (ADG) during the lactation period and BW at weaning (Wolter et al., 2002; Sulabo et al., 2010; Park et al., 2014). Moreover, exposure to pelleted creep feed habituates piglets to consuming a pelleted diet, which can improve average daily feed intake (ADFI) and ADG after weaning (Pluske et al., 2007; Muns and Magowan, 2018). Alternatively, providing supplemental nutrition as liquid milk replacers (LMRs) can reduce variability in creep feed intake among piglets and improve ADG, as well as BW at weaning versus those provided with pelleted creep feed or no creep feed (Kim et al., 2001; Bruininx et al., 2002; van Oostrum et al., 2016). However, milk replacers do not introduce piglets to plant-based protein sources and do not familiarize piglets with solid (pelleted) diets. Moreover, LMRs require specialized feeding equipment and/or increased manual labor versus providing pelleted creep feeds (e.g., for frequently dispensing milk replacer and maintaining feeder cleanliness).

Typically, nursery diets are formulated with highly digestible and expensive protein sources (e.g., whey powder, fishmeal, blood meal) to optimize nutrient absorption and minimize the post-weaning growth lag (Koo et al., 2020). Previous research has demonstrated, however, that pigs fed nursery diets with lower quality protein sources (e.g., soybean meal) can accelerate growth after an initial reduction in ADG and achieve the same BW as those fed high-quality protein sources immediately after weaning (i.e., compensatory growth; Skinner et al., 2014; Huber et al., 2018). However, it is not known whether piglet nutrition during the lactation period can influence the utilization of low-complexity nursery diets. Therefore, the aim of the current study was to determine the effects of creep feed composition and form on pre- and post-weaning pig growth performance and the utilization of low-complexity nursery diets.

## MATERIALS AND METHODS

### Animals and Housing

The experimental protocol was approved by the University of Guelph Animal Care Committee and followed Canadian Council on Animal Care guidelines (CCAC, 2009; AUP #4044). The study was conducted at the University of Guelph Arkell Swine Research Station (Guelph, ON, Canada).

Six hundred seventy-two piglets born to 56 Landrace × Yorkshire first-parity sows (initial BW 190.3 ± 1.33 kg) mated with Duroc boars over seven breeding batches (blocks) were used for the study. Litters were standardized to 12 piglets within 48 h of parturition. Piglets were processed according to standard Arkell Swine Research Station protocol (i.e., weighing, tail docking, ear notching, needle teeth clipping, and iron dextran injection) within 24 h of birth, and at 4 days of age, male pigs were surgically castrated. During the suckling period, piglets were housed with the dam in conventional farrowing crates (183 × 241 cm) with a heating pad (set to 35 °C) in the creep area (Osborne Stansfield nursery pad, Osborne, KS); the creep area was equally distributed on both sides of the farrowing crate. All piglets had unlimited access to water via nipple drinkers. Sows were fed 2 kg of standard lactation diet from day 110 of gestation until farrowing. After parturition, feed was provided in a stepwise manner for 4 days when ad libitum feed allowance was reached. Sow feed intake was recorded weekly. The interval between weaning and breeding was recorded for each sow and sows were only bred when signs of heat were present.

At weaning, six piglets per litter (three castrated males and three females) of median BW were selected to continue the study and were weaned into a nursery pen (112 × 147 cm; one pen per litter) with plastic-coated, expanded metal floors in an environmentally controlled nursery room. Room temperature was 27 °C during the first week after weaning and reduced by 1 °C each week thereafter. Each pen contained a stainless-steel feeder with four head spaces, a nipple drinker, and a toy for enrichment.

### Dietary Treatments and Feeding

Starting at 5 ± 0.3 days of age (initial BW 2.38 ± 0.02 kg) and continuing until weaning at 21 ± 2.1 days of age, litters were provided with one of four creep treatments according to a randomized block design: 1) pelleted commercial creep feed (COM), 2) LMR, 3) pelleted milk replacer (PMR), or 4) no creep feed (NO; *n* = 14). The COM contained corn and fishmeal with no milk products (Floradale Feedmill Ltd., Floradale, ON, Canada), while the LMR (powder) and PMR were formulated with matched levels of net energy (2,903 kcal/kg), crude protein (CP; 22%), crude fat (10%), and standardized ileal digestible (SID) Lys (1.61%), supplied by milk products (Table 1; Grober Nutrition, Cambridge, ON, Canada). The PMR contained 12% corn to facilitate pelleting. The COM and PMR were pelleted (3.5 mm diameter) using a conditioning temperature between 75 and 78 °C. All creep feeds and milk replacer powder were mixed with 1% (wt/wt) brilliant blue dye prior to feeding for visual identification of individual piglets that consumed creep feed via appearance in the feces (Agyekum et al., 2018). The blue dye was included with the milk replacer powder and was coated onto the pelleted diets using canola oil (i.e., 5 mL of oil per 300 g of pellets). The creep feeds and milk replacer were provided in MS Schippers MS Click Feeder Mini (1.5 L capacity; MS Schipper, Lacombe County, AB, Canada). Feeders were placed at the front of the crate, adjacent to the heating pad. Litters that received the PMR or COM creep treatments were given 300 g of feed at 0930 h once daily. Litters that received the LMR creep treatment were given fresh milk replacer twice daily at 0930 h and 1430 h. For the first 7 days of creep treatment, litters provided with LMR were fed daily portions of 1.5 L (250 g of milk replacer powder per liter of warm water), 2 L for the subsequent 4 days, and 3 L thereafter until weaning. Creep feed disappearance was determined daily on a dry matter (DM) basis.

**Table 1.** Calculated and analyzed nutrient contents (as-fed basis) of commercial creep feed (COM), liquid milk replacer (LMR), and pelleted milk replacer (PMR)

Item	COM^1^	LMR^2^	PMR^2^
Guaranteed analysis			
NE, kcal/kg	2,541	2,903	2,903
Crude protein, %	22	22	22
Calcium, %	0.9	0.9	0.9
Phosphorus, %	0.8	0.8	0.8
Total Lys, %	1.7	1.7	1.8
SID Lys, %^3^	1.5	1.6	1.6
Analyzed nutrient content, %			
Dry matter	92.14	93.01	92.84
Crude protein	23.24	23.09	21.38
Calcium	0.78	0.86	0.81
Phosphorus	0.73	0.80	0.80
Potassium	1.17	1.49	1.42
Magnesium	0.12	0.08	0.08
Sodium	0.43	0.57	0.48

^1^Commerical creep feed from Floradale Feed Mill (Floradale, ON, Canada).

^2^LMR (powder) and PMR from Grober Nutrition (Cambridge, ON, Canada).

^3^Standardized ileal digestible.

At weaning, six pigs from each litter that were classified as “eaters” (i.e., produced fecal swabs positive for blue dye at least on days 17 and 21 of age) were selected and placed in a nursery pen (one pen per litter; pen was the experimental unit). Nursery pens were randomly assigned either a HIGH− (contained highly digestible animal protein sources) or LOW− (contained corn and soybean meal as the main protein sources; Table 2) complexity nursery diet according to a 4 × 2 factorial arrangement (i.e., with creep treatment and nursery diet as the two factors; *n* = 7). Nursery diets were fed in a three-phase feeding program with phases I, II, and III fed for 7, 14, and 17 days, respectively, which were formulated to meet estimated nutrient requirements for nursery pigs within each phase (NRC, 2012). The phase I diets contained 0.3% titanium dioxide as an indigestible marker. Phase III also contained 0.2% titanium dioxide to replace corn during the final 10 days of the study.

**Table 2.** Ingredient composition and calculated and analyzed nutrient contents of nursery diets (as-fed basis)^1^

Item	HIGH			LOW		
	Phase I	Phase II	Phase III^2^	Phase I	Phase II	Phase III^2^
Ingredient, %						
Corn	16.8	37.78	49.59	46.42	49.37	47.2
Soybean meal, dehulled	13	16	22	24	34	37
Wheat	—	—	—	10	10	10
Barley	25	25	20	—	—	—
Fat, animal vegetable blend	2.5	2.5	2.5	2.5	2.5	2.5
Herring meal	5	3	—	5	—	—
Blood plasma^3^	4.5	2	—	—	—	—
Blood meal, spray dried	—	2	2	—	—	—
Oat groats	10	—	—	—	—	—
Whey	20	8	—	8	—	—
L-Lysine·HCl	0.35	0.33	0.38	0.47	0.35	0.1
DL-Methionine	0.1	0.16	0.14	0.06	0.11	—
L-Threonine	0.05	0.13	0.14	0.13	0.09	—
L-Tryptophan	—	0.03	0.05	0.02	—	—
Limestone	1	1.02	1.1	1	1.18	1.1
Salt	—	0.2	0.3	0.2	0.4	0.3
Monocalcium phosphate	0.8	1.25	1.2	1.3	1.4	1.2
Vitamin and mineral premix^4^	0.6	0.6	0.6	0.6	0.6	0.6
Titanium dioxide	0.3	—	—	0.3	—	—
Calculated nutrient composition^5^						
NE, kcal/kg	2,588	2,530	2,500	2,557	2,489	2,475
Crude protein, %	21.5	20.4	19.2	21.3	21.9	22.7
Total Lys, %	1.58	1.45	1.34	1.55	1.44	1.34
SID Lys, %^6^	1.40	1.29	1.19	1.38	1.29	1.17
Calcium, %	0.89	0.85	0.81	0.91	0.87	0.85
Phosphorus, %	0.76	0.75	0.67	0.78	0.75	0.73
Analyzed nutrient composition, %^7^						
Crude protein	22.5	20.6	19.9	21.5	22.4	22.0
Calcium	0.80	0.86	0.75	0.76	0.78	0.78
Phosphorus	0.61	0.80	0.65	0.68	0.70	0.67

^1^Dietary treatments: HIGH, nursery diets that contained protein from plant and animal sources; LOW, nursery diets with corn and soybean meal as the main protein sources. Diets were fed for 7, 14, and 17 days in phases I, II, and III, respectively.

^2^Phase III diets contained 0.2% titanium dioxide at the expense of corn during the final 10 days.

^3^AP920; manufactured by APC Nutrition Inc. (Ames, IA).

^4^Provided, per kilogram of diet, 12,000 IU vitamin A as retinyl acetate, 1,299 IU vitamin D3 as cholecalciferol, 48 IU vitamin E as dl-α-tocopherol acetate, 3 mg vitamin K as menadione, 19 mg pantothenic acid, 6 mg riboflavin, 600 mg choline, 2.4 mg biotin, 18 mg Cu from CuSO_4_·5H_2_O, 120 mg Fe from FeSO_4_, 24 mg Mn from MnSO_4_, 126 mg Zn from ZnO, 0.36 mg Se from Na_2_SeO_3_, and 0.6 mg I from KI (DSM Nutritional Products Canada Inc., Ayr, ON, Canada).

^5^Calculated based on the NRC (2012) ingredient values. For Phase III −/+ titanium dioxide values are calculated on a weighted average basis using the number of days each diet was fed.

^6^Standardized ileal digestible.

^7^Phase III diet analyzed nutrients expressed as a weighted average between Phase III −/+ titanium dioxide fed for 7 and 10 days, respectively.

### Experimental Procedures

Backfat and loin depth of sows were measured at the P2 position (6.5 cm from the midline over the last rib) on day 110 of gestation and at weaning using a portable ultrasound machine with a 140 mm linear probe (Agroscan L, ECM Noveko International Inc., Angoulême, France). Individual sow BW was determined within 24 h after parturition and again at weaning.

Individual piglets were weighed upon initiating the creep feed treatments and were weighed and fecal swabbed on 9, 13, 17, and 21 (weaning) days of age to classify individual piglets as creep feed “eaters” or “non-eaters” via the presence of blue dye in the feces. In the nursery period, individual pigs were weighed and per pen feed disappearance was determined weekly to calculate ADG, ADFI, and gain:feed (G:F) for each phase.

Cameras (Canon Vixia RF800; Canon Canada Inc., Brampton, ON, Canada) were installed above each nursery pen and recorded continuously for 48 h after weaning. Video footage was recorded on memory cards (SanDisk Extreme Pro; Western Digital Technologies Inc., Milpitas, CA) and played back on Windows Media Player (Microsoft Corporation, Redmond, WA) with behavior continuously observed. Individual pigs within each pen were uniquely identified with animal marker that was visible in the video recording. The latency to consume the nursery diet was determined for each individual pig; a feeding event was noted when the pig placed its head in the feeder for three or more seconds or was observed chewing (adapted from Pajor et al., 1991).

At weaning (21 days of age), one-week post-weaning (28 days of age), and at the end of the nursery period (59 days of age), two pigs per litter were randomly selected (one castrated male and one female; 14 pigs per treatment) and were euthanized with an intracardiac injection of 3 mL of Euthasol (Virbac, TX; days 21 and 28) or by electrical stunning followed by exsanguination (day 59). Immediately thereafter, the entire gastrointestinal tract (GIT) was excised and full gut and individual (empty) organ weights were measured. Ileal digesta from the last meter of the small intestine was collected on days 28 and 59. Fresh fecal samples were also collected from pens between days 57 and 59 and from the descending colon during dissection. Both ileal digesta and fecal samples were pooled by pen. Ileal and fecal samples were stored at −20 °C until freeze drying and then were ground and stored at 4 °C until further analysis.

### Nutrient Analysis

Subsamples of each creep feed and nursery diet were collected weekly and combined within phase (for nursery diets) and were finely ground using a coffee grinder (Custom Grind Coffee Grinder, Hamilton Beach Brands Canada Inc., Belleville, ON, Canada). Ground samples were analyzed for DM (AOAC 2005; method 930.15), CP (AOAC 2005; method 968.06), calcium, phosphorus, potassium, and magnesium using inductively coupled plasma mass spectrophotometry (AOAC, 2005; method 985.01; creep diets only) and sodium using inductively coupled plasma-optical emission spectrometry (AOAC, 2005; method 2011.14; creep diets only; Agrifood Laboratories, Guelph, ON, Canada).

Freeze-dried ileal and fecal samples and nursery diet composite samples (phases I and III only) underwent analyses for DM (AOAC, 2005; method 930.15), ash (AOAC, 2005; method 942.05), and nitrogen (via combustion; LECO-FP 828 analyzer, LECO Instruments Ltd, Mississauga, ON, Canada). Titanium content was determined according to Myers et al. (2004) with minor adaptations (digestion for 24-h at 120 °C in 10 mL tubes and addition of H_2_O_2_ after precipitate settled in 100 mL volumetric flasks) and absorbance of standards and samples were measured by spectrophotometry at 408 nm (Epoch 2, BioTek Instruments Inc., Winooski, VT). The gross energy (GE) of nursery diet composite samples and fecal samples was determined via a bomb calorimeter (IKA Calorimeter System C 5000; IKA Works Inc., Wilmington, NC). For all analyses, diet samples were analyzed in quadruplicate and ileal and fecal samples analyzed in triplicate.

### Calculations and Statistical Analysis

Apparent ileal digestibility (AID) of organic matter (OM) and CP and apparent total tract digestibility (ATTD) of OM and GE were calculated according to Fuller et al. (2012). Feed costs for the creep feed and nursery treatments were calculated per pig (using per pen ADFI divided by the number of pigs in a pen) and per kilogram of pig BW at weaning and exit from the nursery, respectively, using current commodity prices. The statistical analysis for lactating sow performance (changes in BW, back fat, and loin depth and weaning-to-estrus interval) and suckling and nursery pig growth performance (ADG, ADFI, G:F, relative organ weights) and feed costs were conducted using the GLIMMEX procedure of SAS (University Edition; SAS Inst. Inc., Cary, NC) with either creep feed treatment as the main effect (prior to weaning) or creep feed treatment, nursery diet, and the interaction between creep feed treatment and nursery diet as the main effects (after weaning). The interaction between creep feed treatment and nursery diet was not significant apart from pig BW during the nursery period and for overall nursery ADG, therefore, only the main effects of creep feed treatment and nursery diet are presented for all other outcomes. Sow and litter (prior to weaning) or pen (after weaning) were the experimental units. Block (breeding batch) was considered a random effect and initial BW (i.e., at the start of creep feeding) was used as a covariate for offspring growth performance outcomes. The proportion of piglets that had blue fecal swabs on each sampling day was analyzed as a binary distribution to determine the odds of each piglet having a blue fecal swab. In all analyses, the degrees of freedom were calculated with Kenward-Roger’s adjustment for repeated measures and outliers were detected using the univariate procedure. Mean comparisons were conducted using Tukey-Kramer test to separate means. Probability (*P*)-values of less than 0.05 were considered significant, and 0.05 ≤ *P* ≤ 0.10 were considered tendencies.

## RESULTS

The analyzed and calculated nutrient contents for the creep feed treatments were comparable. The analyzed and calculated nutrient contents were also generally comparable for the nursery diets. The exception was for the phase I diets where the analyzed Ca content was 20% lower versus calculated for the LOW diet and the analyzed P contents were 15% and 24% lower versus calculated for the LOW and HIGH diets, respectively. During the suckling period, 28%, 14%, 8%, and 7% of individual piglets that received the LMR, PMR, COM, and NO creep feed treatments, respectively, were treated for diarrhea, though there was no difference in mortality (approximately 2%) during the creep feeding period among treatments (*n* = 14). In addition, neither creep nor nursery treatment affected mortality in the nursery period (approximately 1%; *n* = 7).

### Lactation Performance

Initial BW for sows among all creep treatments were not different (Table 3). Creep feed treatment did not influence changes in sow BW, backfat depth, loin depth, or ADFI during the lactation period or the wean-to-estrus interval. Initial BW of piglets and litter size were not different among creep feed treatments. The ADG over the creep feed treatment period was greater for piglets that received the COM and LMR treatments versus those that received the PMR treatment (*P* < 0.05); intermediate values were observed for piglets that received the NO treatment. The average daily creep feed disappearance (DM-basis) was greater for piglets that received the LMR versus the COM and PMR treatments (*P* < 0.05), which were not different. Piglets that received the LMR had greater BW at weaning than all other creep treatment groups (*P* < 0.05), piglets that received the COM had greater BW at weaning than those that received the PMR (*P* < 0.05), and piglets that received the NO treatment had intermediate BW at weaning versus the COM and PMR groups. The estimated cost of creep feed per pig and cost of creep feed per kilogram of BW at weaning were greater for piglets that received LMR versus those that received COM and PMR (*P* < 0.001), which were not different.

**Table 3.** Effect of creep feed form and composition on sow and piglet performance during lactation

	Treatment^1^					
	COM	LMR	PMR	NO	SEM^2^	*P*-value
No.^3^	14	14	14	14		
Sow performance						
Initial body weight, kg	183.8	192.5	196.3	188.8	7.1	0.623
Change in BW, kg^4^	−10.0	−8.8	−7.8	−9.9	3.2	0.443
Change in backfat, mm^5^	−2.2	−2.5	−2.7	−2.7	0.7	0.916
Change in loin depth, mm^5^	−1.2	−3.5	−4.2	−4.5	1.3	0.250
Average daily feed intake, kg^6^	6.9	6.4	6.5	6.9	0.5	0.909
Estrus interval, days	4.9	4.9	5.0	5.2	0.3	0.857
Piglet performance						
Litter size	11.9	11.7	11.6	11.9	0.2	0.708
Initial BW, kg^7^	2.45	2.34	2.35	2.36	0.08	0.268
Averaged daily gain, g	268^a^	280^a^	248^b^	260^ab^	10	<0.001
Average daily creep feed disappearance, g^8^	132^b^	452^a^	127^b^	—	18	<0.001
Weaning BW, kg	6.02^b^	6.33^a^	5.66^c^	5.92^bc^	0.14	<0.001
Creep feed cost, $/pig	0.31^b^	2.33^a^	0.38^b^	—	0.06	<0.001
Creep feed cost, $/kg BW at weaning	0.05^b^	0.39^a^	0.07^b^	—	0.04	<0.001

^1^Creep feed treatments: commercial creep feed from Floradale Feed Mill (Floradale, ON, Canada; micropellets; COM), liquid milk replacer (LMR), and pelleted milk replacer (micropellets; PMR) from Grober Nutrition (Cambridge, ON, Canada), or no creep feed offered (NO). Creep feed treatments were implemented at 5.4 ± 0.3 days of age and until weaning (21.3 ± 2.1 days of age).

^2^Maximum value for the standard error of the means.

^3^Number of sows or litters.

^4^Sow BW was measured within 24 h of farrowing and at weaning (21 ± 2.1days).

^5^Backfat and loin depth were measured on day 110 of gestation and at weaning.

^6^Sows were fed a commercial lactation diet. Feed intake was recorded between the start of the creep feed treatment until weaning.

^7^Initial piglet BW was recorded upon initiating creep feed treatments.

^8^Average daily creep feed disappearance per litter (DM-basis) for the creep feeding period.

^a–c^Within a row, means without a common superscript differ, *P* < 0.05.

The average daily creep feed disappearance between each fecal swabbing period and the occurrence of piglets with blue feces (“eaters”) were greater for piglets that received the LMR treatment versus other groups (*P* < 0.05) but were generally not different between piglets that received the COM and PMR treatments (Table 4). Only on 13 days of age did piglets that received the PMR treatment have a greater occurrence of blue feces than those that received the COM treatment (*P* < 0.05) and between days 14 and 15, piglets that received the PMR had lower average daily creep feed disappearance than those that received the COM treatment (*P* < 0.05).

**Table 4.** Effects creep feed form and composition on feed disappearance and the percentage of pigs consuming creep feed^1^

	Treatment^1^				
Item	COM	LMR	PMR	SEM^2^	*P*-value
No.^3^	14	14	14		
Average daily creep feed disappearance, g/pig^4^					
Day 5 to 9	4.3^b^	18.8^a^	4.3^b^	0.8	<0.001
Day 10 to 13	8.4^b^	28.2^a^	6.7^b^	1.6	<0.001
Day 14 to 15	11.7^b^	36.1^a^	9.2^c^	2.3	<0.001
Day 16 to 17	12.4^b^	48.8^a^	14.8^b^	2.9	<0.001
Day 18 to 21	16.4^b^	55.7^a^	16.6^b^	1.7	<0.001
Blue-positive feces, %^5^					
Day 9	10.9^b^	63.8^a^	16.1^b^	3.8	<0.001
Day 13	51.5^c^	86.9^a^	65.4^b^	3.9	<0.001
Day 15	64.9^b^	86.3^a^	73.2^b^	3.6	<0.001
Day 17	70.1^b^	91.3^a^	75.0^b^	3.5	<0.001
Day 21	77.3^b^	96.9^a^	85.3^b^	3.3	<0.001

^1^Creep feed treatments: commercial creep feed from Floradale Feed Mill (Floradale, ON, Canada; micropellets; COM), liquid milk replacer (LMR), and pelleted milk replacer (micropellets; PMR) from Grober Nutrition (Cambridge, ON, Canada), or no creep feed offered (NO). Creep feed treatments were implemented at 5.4 ± 0.3 days of age and until weaning (21.3 ± 2.1 days of age).

^2^Maximum value for the standard error of the means.

^3^Number of litters evaluated.

^4^Average daily creep feed disappearance per pig (DM-basis).

^5^Brilliant blue dye was included with the creep diets (1%, as-fed) and presence in feces was determined via visual inspection. Percent of pigs with blue feces within a litter.

^a–c^Within a row, means without a common superscript differ, *P* < 0.05.

### Nursery Growth Performance

**Figure 1.**
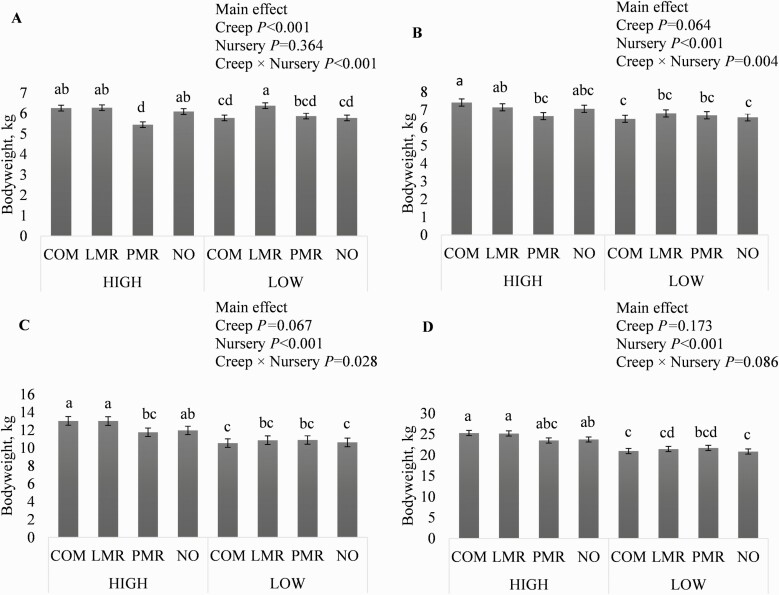
Interaction between creep and nursery feed treatments on bodyweight at (A) weaning (21 days of age), (B) 28 days of age, (C) 42 days of age, and (D) 59 days of age. Creep feed treatments: commercial creep feed (COM), liquid milk replacer (LMR), pelleted milk replacer (PMR), or no creep feed offered (NO). Nursery feed treatments: HIGH, nursery diets that contained protein from plant and animal sources; LOW, nursery diets with corn and soybean meal as the main protein sources. Values are LSmeans ± SEM, *n* = 7. LSmeans without a common letter differ, *P* < 0.05.

After weaning, creep feed treatment nor nursery diet complexity influenced the latency to consuming the first meal or apparent daily feed intake during the first 48 h (Table 5). Initial BW upon entering the nursery (21 days of age) was influenced by the interaction of creep and nursery feed treatments (*P* < 0.001; Figure 1, Panel A) and the main effect of creep treatment (*P* < 0.001; Table 6). Within the HIGH nursery diet treatment, pigs that received PMR during the creep feeding period had lower BW on day 21 versus pigs that received all other creep treatments (*P* < 0.05) and within the LOW nursery diet treatment, pigs that received LMR during the creep feeding period had greater BW on day 21 versus pigs that received all other creep treatments (*P* < 0.05). On day 28 (end of phase I), pig BW was influenced by the interaction of creep and nursery feed treatments (*P* < 0.05) and the main effects of creep (tendency; *P* = 0.064) and nursery feed treatments (*P* < 0.001; Figure 1; Panel B). Within the HIGH nursery diet treatment, pigs that received PMR during the creep feeding period had lower BW versus pigs fed COM (*P* < 0.05), while the BW of pigs that received LMR and NO were intermediate. Within the LOW nursery diet treatment, there were no differences in BW among creep feed treatments. On day 42 (end of phase II), pig BW was influenced by the interaction of creep and nursery feed treatments (*P* < 0.05) and the main effects of creep (tendency; *P* = 0.067) and nursery feed treatments (*P* < 0.001; Figure 1; Panel C). Within the HIGH nursery diet treatment, pigs that received PMR during the creep feeding period had lower BW versus pigs fed COM and LMR (*P* < 0.05), while the BW of pigs that received NO were intermediate. Within the LOW nursery diet treatment, there were no differences in BW among creep feed treatments. On day 59 (end of phase III), pig BW tended to be influenced by the interaction of creep and nursery feed treatments (*P* = 0.086) and the main effects of creep (*P* = 0.065) and nursery feed treatment (*P* < 0.001) but for either nursery diet treatment there were no differences in BW among pigs fed the various creep feed treatments (Figure 1; Panel D). On days 28, 42, and 59, BW were less for pigs that received the LOW versus the HIGH nursery diet (*P* < 0.001; Table 6).

**Table 5.** The effect of creep feed form and composition on latency between weaning and first meal and nursery feed intake for the first 2 days post-weaning

	Creep Treatment^1^					Nursery Treatment^2^			*P*-value^3^	
	COM	LMR	PMR	NO	SEM^4^	HIGH	LOW	SEM^4^	CREEP	NURSERY
No.^5^	14	14	14	14		28	28			
Latency, h^6^	13.1	8.4	8.5	7.8	2.1	10.6	8.3	1.6	0.097	0.117
Apparent feed intake, g/pig/day^7^	253	285	253	237	30	261	253	20	0.584	0.771

^1^Creep feed treatments: commercial creep feed from Floradale Feed Mill (Floradale, ON, Canada; micropellets; COM), liquid milk replacer (LMR), and pelleted milk replacer (micropellets; PMR) from Grober Nutrition (Cambridge, ON, Canada), or no creep feed offered (NO). Creep feed treatments were implemented at 5.4 ± 0.3 days of age and until weaning (21 ± 2.1 days of age).

^2^Nursery feed treatments: HIGH, nursery diets that contained protein from plant and animal sources; LOW, nursery diets with corn and soybean meal as the main protein sources. Diets were fed for 7, 14, and 17 days in phases I, II, and III, respectively.

^3^ *P*-values for the main effects of creep (CREEP) and nursery dietary treatment (NURSERY).

^4^Maximum value for the standard error of the means.

^5^Number litters evaluated.

^6^Latency between weaning and eating was measured for each pig within each pen. A feeding bout was noted when the pig’s head was in the feeder for three or more seconds or was observed chewing.

^7^Apparent feed intake for pigs during the first 2 days post-weaning.

**Table 6.** The effect of creep feed composition and form and nursery diet complexity on growth performance of pigs after weaning

	Creep treatment^1^					Nursery treatment^2^			*P*-value^3^	
	COM	LMR	PMR	NO	SEM^4^	HIGH	LOW	SEM^4^	CREEP	NURSERY
No.^5^	14	14	14	14		28	28			
Body weight, kg^6^										
Day 21	6.02^b^	6.33^a^	5.66^c^	5.92^bc^	0.14	6.02	6.00	0.08	<0.0001	0.364
Day 28	6.96^xy^	6.97^x^	6.66^y^	6.80^xy^	0.20	7.07	6.63	0.19	0.064	<0.001
Day 42	11.79	11.94	11.33	11.30	0.48	12.45	10.74	0.40	0.067	<0.001
Day 59	23.16^xy^	23.45^x^	22.47^xy^	22.25^y^	0.62	24.50	21.24	0.55	0.065	<0.001
ADG, g/kg of BW										
Phase I	15.7	13.6	17.7	14.5	1.8	19.0	11.7	1.5	0.166	<0.001
Phase II	29.0^xy^	29.9^x^	29.9^xy^	28.0^y^	0.8	30.8	27.5	0.6	0.077	<0.001
Phase III	27.3	26.9	27.6	27.4	0.7	27.3	27.3	0.6	0.425	0.808
Overall^7^	26.2	26.3	27.2	26.1	0.4	27.7	25.1	0.4	0.154	<0.001
ADFI, g/kg of BW										
Phase I	24.2^ab^	25.9^a^	23.9^ab^	22.2^b^	3.1	25.8	22.7	3.1	0.044	<0.001
Phase II	35.1^y^	36.3^y^	38.4^x^	38.2^x^	1.6	38.0	36.1	1.4	0.036	0.044
Phase III	36.5^x^	33.5^y^	35.9^xy^	36.2^xy^	1.1	35.2	35.8	0.9	0.046	0.462
Overall	34.2	34.1	35.7	35.3	1.3	35.2	34.4	0.7	0.328	0.326
G:F										
Phase I	0.78	0.62	0.80	0.59	0.13	0.77	0.62	0.12	0.093	0.031
Phase II	0.88	0.85	0.80	0.79	0.05	0.86	0.80	0.04	0.071	0.070
Phase III	0.77	0.81	0.79	0.78	0.02	0.79	0.78	0.02	0.541	0.685
Overall	0.79	0.78	0.78	0.76	0.03	0.81	0.75	0.02	0.764	0.001
Feed cost, $/pig										
Nursery	14.42	14.09	14.19	14.31	0.57	16.42	12.08	0.55	0.757	<0.001
Creep + nursery	14.75^b^	16.42^a^	14.58^b^	14.34^b^	0.59	17.17	12.87	0.56	<0.001	<0.001
Total feed cost, $/kg exit BW^8^	0.65^b^	0.71^a^	0.65^b^	0.65^b^	0.02	0.71	0.62	0.02	0.001	<0.001

^1^Creep feed treatments: commercial creep feed from Floradale Feed Mill (Floradale, ON, Canada; micropellets; COM), liquid milk replacer (LMR), and pelleted milk replacer (micropellets; PMR) from Grober Nutrition (Cambridge, ON, Canada), or no creep feed offered (NO). Creep feed treatments were implemented at 5.4 ± 0.3 days of age and until weaning (21.3 ± 2.1 days of age).

^2^Nursery feed treatments: HIGH, nursery diets that contained protein from plant and animal sources; LOW, nursery diets with corn and soybean meal as the main protein sources. Diets were fed for 7, 14, and 17 days in phases I, II, and III, respectively.

^3^ *P*-values for the main effects of creep (CREEP) and nursery dietary treatment (NURSERY).

^4^Maximum value for the standard error of the means.

^5^Number litters evaluated.

^6^BW was influenced by the interaction of creep and nursery feed treatments; Day 21 *P* < 0.001, Day 28 *P* < 0.01, Day 42 *P* < 0.05, Day 59 *P* = 0.068; see Figure 1.

**Figure 2.**
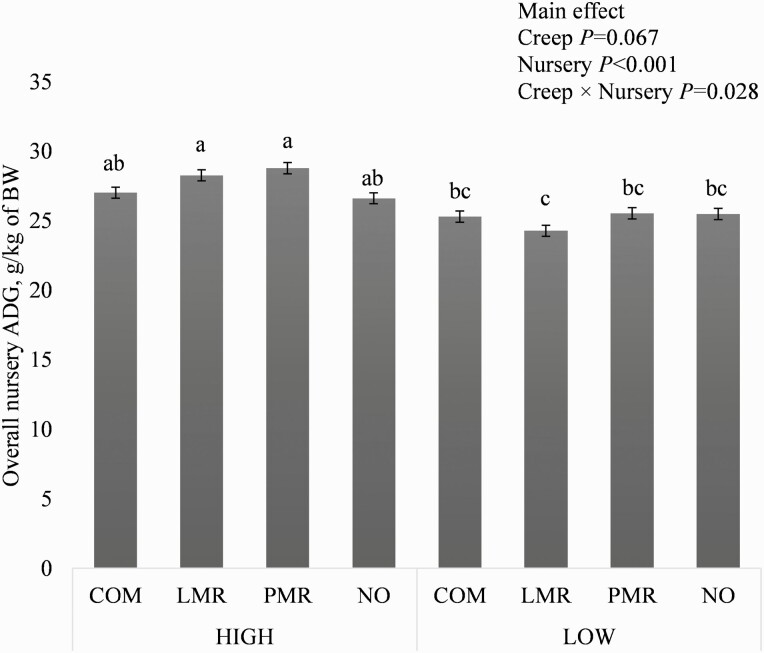
Interaction between creep and nursery feed treatments on ADG, g/kg of live BW throughout the nursery period. Creep feed treatments: commercial creep feed (COM), liquid milk replacer (LMR), pelleted milk replacer (PMR), or no creep feed offered (NO). Nursery feed treatments: HIGH, nursery diets that contained protein from plant and animal sources; LOW, nursery diets with corn and soybean meal as the main protein sources. Values are presented as LSmeans ± SEM, *n* = 7. LSmeans without a common letter differ, *P* < 0.05.

^7^Overall ADG was influenced by the interaction of creep and nursery feed treatments; *P* < 0.05; see Figure 2.

^8^Sum of creep and nursery diets consumed per pig divided by nursery exit BW.

^a–c^Within a row, means without a common superscript differ, *P* < 0.05.

^x,y^Within a row, means without a common superscript differ, 0.05 ≤ *P* ≤ 0.10.

The per-phase ADG, ADFI, and G:F were not influenced by the interaction of creep and nursery treatments, therefore, only the main effects are presented. In phase I, ADG and G:F were not influenced by the main effect of creep feed treatment, but were less for pigs fed LOW versus HIGH complexity nursery diets (*P* < 0.05; Table 6). In phase I, ADFI was greater for pigs that received LMR versus those that received NO during the suckling period (*P* < 0.05), while intermediate values were observed for pigs that received the COM and PMR. In phase I, ADFI was less for pigs fed LOW versus HIGH complexity diets (*P* < 0.001). In phase II, ADG tended to be greater for pigs that received LMR versus those that received NO during the suckling period (*P* = 0.098), while intermediate values were observed for pigs that received the COM and PMR. In phase II, ADFI tended to be greater for pigs that received PMR and NO versus those that received COM during the suckling period (*P* = 0.079), while pigs that received LMR had intermediate ADFI. The G:F in phase II tended to be influenced by creep feed treatment (*P* = 0.071). In phase II, ADG, ADFI, and G:F, were less for pigs that received LOW versus HIGH nursery diets (*P* < 0.001, *P* < 0.05, and *P* = 0.070 for ADG, ADFI, and G:F, respectively). In phase III, ADFI tended to be greater for pigs that received COM versus LMR in the suckling period (*P* = 0.058), intermediate values were observed for PMR and NO; ADG and G:F were not influenced by creep feed treatment and ADG, ADFI, and G:F were not influenced by nursery treatment.

Overall (between 21 and 59 days of age), ADG was influenced by the interaction between creep and nursery treatments (*P* < 0.05; Figure 2). Pigs that received LMR or PMR during the creep feeding period and the HIGH nursery diet had greater ADG than all pigs fed the LOW nursery diet, regardless of creep feeding treatment (*P* < 0.05), while pigs fed COM-HIGH, NO-HIGH, COM-LOW, PMR-LOW, and NO-LOW were intermediate. Creep treatment provided during the suckling period did not influence overall ADFI and G:F in the nursery, ADG and G:F were less for pigs that received LOW versus HIGH nursery diets (*P* < 0.001), and ADFI was not affected by nursery diet treatment (Table 6). Feed cost per pig during the nursery period was not influenced by creep feeding treatment during the suckling period but was less for pigs that received the LOW versus HIGH nursery diet (*P* < 0.001). Cumulative feed cost (i.e., during the creep feeding and nursery periods) per pig and per kilogram of nursery exit BW were greater for pigs that received LMR versus all other creep feeds (*P* < 0.05) and less for pigs that received the LOW versus HIGH nursery diet (*P <* 0.001).

### Relative Organ Weights After Weaning

On day 21 of age, live BW and relative weights of the GIT segments and liver were not affected by creep feed treatment (Table 7). On days 28 and 59, live BW was influenced by the interaction of creep feed and nursery treatments, but the interaction was not significant for any other outcome (data not shown). On day 28, live BW was less for pigs that received LOW versus HIGH nursery treatment (*P* < 0.05) but relative GIT segments were not affected by creep feed or nursery treatment, and relative liver weight tended to be influenced by creep feed treatment (*P* = 0.089). On day 59, live BW was influenced by creep feed treatment (*P* < 0.05) and was less for pigs that received LOW versus HIGH nursery diets (*P* < 0.001). The relative full gut weight (g/kg of BW) was greater and small intestine tended to be greater for pigs that received PMR versus those that received LMR during the suckling period (*P* < 0.05 and *P* = 0.051, respectively), while intermediate values were observed for NO and COM. Relative full gut and small intestine weights were also greater for pigs that received LOW versus HIGH nursery treatment (*P* < 0.01). Relative empty stomach weight was greater for pigs that received PMR versus COM during the suckling period (*P* < 0.05), while intermediate values were observed for LMR and NO; relative empty stomach weight was not influenced by nursery treatment. Relative large intestine weight was not influenced by creep feed treatment but was greater for pigs that received the LOW versus the HIGH nursery diet (*P* < 0.05). Relative liver weight was greater for pigs that received PMR versus those that received NO creep feed during the suckling period (*P* < 0.05), while intermediate values were observed for COM and LMR. Relative liver weight was greater for pigs that received the LOW versus HIGH nursery diet (*P* < 0.001).

**Table 7.** The effect of creep feed composition and form and nursery diet complexity on relative organ weights of pigs after weaning

	Creep treatment^1^					Nursery treatment^2^			*P*-value^3^	
	COM	LMR	PMR	NO	SEM^4^	HIGH	LOW	SEM^4^	CREEP	NURSERY
No.^5^	14	14	14	14		28	28			
Live body weight, kg										
Day 21	6.32	6.29	6.00	6.58	0.49	—	—	—	0.711	—
Day 28†	7.17	7.17	6.77	6.88	0.23	7.36	6.64	0.16	0.495	0.002
Day 59†	23.64^y^	23.80^y^	21.50^x^	22.76^xy^	0.72	24.80	21.06	0.54	0.049	<0.001
Full gut, g/kg of BW										
Day 21	68.8	77.0	75.4	76.2	8.6	-	-	-	0.631	-
Day 28	157.5	272.4	154.9	204.8	48.4	209.2	185.6	34.6	0.292	0.629
Day 59	164.7^ab^	158.2^b^	176.5^a^	160.7^b^	5.9	159.5	170.7	2.9	0.014	0.008
Stomach, g/kg of BW										
Day 21	5.4	5.4	5.5	5.2	0.5	—	—	—	0.608	—
Day 28	7.1	6.9	7.4	7.3	0.2	7.2	7.2	0.2	0.326	0.919
Day 59	7.2^b^	7.3^ab^	7.8^a^	7.7^ab^	0.2	7.5	7.4	0.2	0.017	0.605
Small intestine, g/kg of BW										
Day 21	35.0	38.1	37.0	35.7	2.7	—	—	—	0.534	—
Day 28	46.7	48.5	47.1	46.8	0.4	47.8	46.8	1.4	0.751	0.448
Day 59	53.1^xy^	51.2^y^	55.4^x^	52.4^xy^	1.2	49.5	56.5	0.8	0.071	<0.001
Large intestine, g/kg of BW										
Day 21	9.1	10.0	9.9	9.7	1.1	—	—	—	0.562	—
Day 28	18.1	17.0	17.1	16.7	0.4	16.9	17.5	0.7	0.596	0.401
Day 59	21.6	21.0	21.8	21.1	0.8	20.7	22.1	0.4	0.680	0.010
Liver, g/kg of BW										
Day 21	24.2	24.7	24.6	23.4	1.4	—	—	—	0.443	—
Day 28	24.8	26.5	24.5	24.4	0.7	24.8	25.3	0.5	0.089	0.499
Day 59	30.2^ab^	30.1^ab^	31.7^a^	29.3^b^	0.9	28.6	32.1	0.5	0.067	<0.001

^1^Creep feed treatments: commercial creep feed from Floradale Feed Mill (Floradale, ON, Canada; micropellets; COM), liquid milk replacer (LMR), and pelleted milk replacer (micropellets; PMR) from Grober Nutrition (Cambridge, ON, Canada), or no creep feed offered (NO). Creep feed treatments were implemented at 5.4 ± 0.3 days of age and until weaning (21.3 ± 2.1 days of age).

^2^Nursery feed treatments: HIGH, nursery diets that contained protein from plant and animal sources; LOW, nursery diets with corn and soybean meal as the main protein sources. Diets were fed for 7, 14, and 17 days in phases I, II, and III, respectively.

^3^ *P*-values for the main effects of creep (CREEP) and nursery dietary treatment (NURSERY).

^4^Maxiumum value for the standard error of the means.

^5^Number litters evaluated.

^a,b^Within a row, means without a common superscript differ, *P* < 0.05.

^x,y^Within a row, means without a common superscript differ, 0.05 ≤ *P* ≤ 0.10.

†Significant interaction between the main effects of CREEP and NURSERY, *P* < 0.05.

### Apparent Nutrient and Energy Digestibility After Weaning

In phase I, the AID of OM tended to be less for pigs that received LOW versus HIGH nursery diets (*P* = 0.076; Table 8), but was not influenced by creep feed treatment. In phases I and III, the AID of CP and the ATTD (phase III only) of GE and OM were not influenced by creep feed or nursery diet treatments.

**Table 8.** The effect of creep feed composition and form and nursery diet complexity on apparent ileal digestibility (AID) and apparent total tract digestibility (ATTD) of nutrients and energy after weaning

	Creep^1^					Nursery^2^			*P*-value^3^	
	COM	LMR	PMR	NO	SEM^4^	HIGH	LOW	SEM^4^	CREEP	NURSERY
No.^5^	14	14	14	14		28	28			
AID, %										
Phase I										
Organic matter	66.4	63.6	68.3	67.8	3.8	68.8	64.2	2.5	0.574	0.076
Crude protein	53.9	53.2	58.1	54.1	5.5	56.1	53.6	4.8	0.745	0.463
Phase III										
Organic matter	64.2	63.0	61.7	61.7	2.3	63.2	62.1	2.0	0.792	0.583
Crude protein	74.8	72.1	72.6	72.0	1.8	74.5	71.6	1.3	0.583	0.108
ATTD										
Phase III										
Organic matter, %	77.5	81.5	81.3	80.0	1.5	79.8	80.3	1.4	0.199	0.699
GE, %	78.4	82.3	80.8	82.5	1.7	80.6	81.5	1.3	0.180	0.536
DE, kcal/kg	3,548	3,723	3,653	3,729	73	3,627	3,700	52	0.199	0.255

^1^Creep feed treatments: commercial creep feed from Floradale Feed Mill (Floradale, ON, Canada; micropellets; COM), liquid milk replacer (LMR), and pelleted milk replacer (micropellets; PMR) from Grober Nutrition (Cambridge, ON, Canada), or no creep feed offered (NO). Creep feed treatments were implemented at 5.4 ± 0.3 days of age and until weaning (21.3 ± 2.1 days of age).

^2^Nursery feed treatments: HIGH, nursery diets that contained protein from plant and animal sources; LOW, nursery diets with corn and soybean meal as the main protein sources. Diets were fed for 7, 14, and 17 days in phases I, II, and III, respectively.

^3^ *P*-values for the main effects of creep (CREEP) and nursery dietary treatment (NURSERY).

^4^Maximum value for the standard error of the means.

^5^Number litters evaluated.

## DISCUSSION

The purpose of the current study was to determine the effects of creep feed composition and form on pig growth performance pre- and post-weaning and the utilization of low-complexity nursery diets. Creep feed form was the most important factor influencing DM intake and growth during the suckling period since LMR was consumed in greater quantities than PMR on a DM basis, despite having similar ingredient and nutrient compositions, which resulted in improved pre-weaning ADG and BW at weaning. Piglets fed LMR also had greater DM intake and BW at weaning than those fed a commercial (pelleted) creep feed. Moreover, BW at weaning was less for piglets that received PMR versus piglets that received LMR and COM and not different from piglets that received no creep feed. This was despite PMR-fed piglets exhibiting ADFI and appearance of blue feces not different from COM-fed piglets, which could be due to poor pellet quality of the PMR (feed wastage) and binary classification (i.e., yes/no) versus quantification of blue dye appearance in feces, respectively. Finally, the greater BW at weaning for LMR-fed piglets did not translate to improved growth performance in the nursery period, regardless of nursery diet complexity. This is in contrast to the work of others that demonstrated improvements in nursery (e.g., Kim et al., 2001; Sulabo et al., 2010) and grower/finisher (Wolter et al., 2002) growth performance when pigs were offered milk replacer during the suckling period. In the current study, the familiarity with consuming feed (liquid or pellets) prior to weaning also did not reduce the latency to access nursery diet or apparent feed intake immediately after weaning. In addition, the use of creep feeds did not influence or minimize sow BW change, back fat, and loin depth loss, or feed intake during the lactation period. Therefore, the provision of creep feed did not rescue sow BW loss during lactation or have extended benefits for the pigs in the post-weaning period.

It is noted that the amount of LMR provided per piglet was limited by the reservoir capacity of the feeders, as well as labor requirements for mixing and delivering the milk replacer. By the end of the suckling period, the amount of LMR provided was capped at 3.0 L (as-fed; 0.75 kg of milk replacer powder) per litter per day. Furthermore, the ADG between days 18 and 21 of age was not different among creep feed treatment groups. If LMR was provided ad libitum, it is possible that benefits for the sow during lactation and the piglets after weaning would be apparent. Previous studies only noted a reduction in sow backfat loss when each piglet consumed an additional 10 g per day of the milk replacer powder than what was observed in the current study (Novotni-Dankó et al., 2015). Therefore, it is suggested that a more frequent feeding schedule or a liquid feeding system should be used to maximize litter LMR intake.

In the current study, pigs fed low complexity nursery diets that contained corn and soybean meal as the main protein sources had lower ADG, ADFI, and G:F in the early nursery period and were unable to exhibit compensatory growth to achieve BW not different from those fed high complexity nursery diets by the end of the nursery period. This is in contrast to the results of others (e.g., Huber et al., 2018; Lafleur Larivière et al., 2021), though in some cases, compensatory growth was not achieved until the end of the grower period (e.g., Skinner et al., 2014). It is noteworthy that the piglets used in the current study were exclusively from first parity sows and had lower BW at weaning (regardless of creep feed treatment) than the aforementioned studies that demonstrated compensatory growth for pigs fed low complexity nursery diets. Typically, offspring from first parity sows have poorer growth performance after weaning and until market weight, which is partly attributed to lighter birth weights and reduced immunoglobulin levels in colostrum and milk of first compared to multi-parity sows (Miller et al., 2012; Piñeiro et al., 2019). However, previous work demonstrated that gilt progeny not provided with milk replacer in the suckling period achieved BW not different from gilt progeny that were provided milk replacer by 10 weeks of age (Miller et al., 2012). It is unknown whether the lower birth and weaning weights of first-parity offspring (vs. offspring from multiparous sows) influence the ability to achieve compensatory growth after a post-weaning nutritional challenge.

Providing supplemental nutrients during the suckling period has been previously shown to benefit intestinal morphology in terms of greater villus height and reduced crypt depth for pigs four days after weaning (Zijlstra et al., 1996), which can improve nutrient absorption (Cera et al., 1988). Thus, it was hypothesized that creep feed or milk replacer intake during the suckling period would improve nutrient and energy digestibility in the nursery period. In the current study, there were no differences in AID or ATTD of OM, CP (AID only), or GE (ATTD only) related to creep feed treatment either at 28 (end of nursery phase I) or 59 (end of nursery phase III) days of age. Conversely, the AID of OM and CP at 28 days of age were 7% and 5% less, respectively, for pigs that received the LOW versus HIGH nursery diet. Therefore, it appeared that the low complexity diet (i.e., with protein supplied mainly by corn and soybean meal) was less digestible by the small intestine early in the nursery period than the high complexity diet (i.e., with highly digestible animal protein sources). Thereafter, and including ATTD, component digestibility was not influenced by nursery diet treatment, despite pigs that received the LOW nursery diet having greater relative visceral organ weights by 59 days of age. Indeed, previous work has shown that pigs develop larger visceral organs to utilize less digestible diets (Pluske et al., 2003). However, visceral organs also increase energy requirements for maintenance and reduce carcass value at slaughter, both of which negatively impact profitability (Nyachoti et al., 2000). Determining whether the greater visceral organ weights were maintained until market weight was beyond the scope of the study, but it is important that both creep and nursery feeding regimens promote adequate development of the GIT and nutrient utilization for protein deposition in the carcass, without disproportionally increasing visceral mass.

In the current study, LMR was the most expensive creep feeding option in terms of cost per pig and cost per kilogram BW at weaning, which was largely driven by differences in apparent intake among the creep feed treatments. Moreover, these cost estimates do not account for the additional labor or specialized feeding systems required to provide liquid creep feeds. Despite heavier BW at weaning, providing LMR did not translate into more efficient use of the nursery diets and subsequent feed cost savings. Conversely, and regardless of creep feeding treatment, providing a low complexity nursery diet resulted in approximately $4.30 feed cost savings per pig and $0.10 per kilogram of (nursery) exit BW. Therefore, the improvement in growth performance for pigs fed the high complexity nursery diet was not proportional to the greater feed cost.

## CONCLUSION

In summary, providing LMR during the suckling period increased piglet BW at weaning, with no apparent benefit for the sow. Furthermore, supplying creep feed during the suckling period had limited impact on the utilization of nursery diets but pigs that received high complexity nursery diets had improved growth performance, particularly early after weaning. However, regardless of creep feeding regimen, low complexity diets are a means to reduce nursery feed costs. Therefore, based on the current study, nursery diets influence post-weaning growth performance to a greater extent than creep feeding regimens. Additional research is required to determine the effects of creep feeding strategy and nursery diet complexity for primiparous offspring on compensatory growth during the grower/finisher phase, in addition to days-to-market and carcass quality.

## References

[CIT0001] Agyekum, A., D. Beaulieu, J. Brown, and Y. Seddon. 2018. Creep feeding can aid post-weaning feed intake and piglet growth. National Hog Farmer. Available from https://www.nationalhogfarmer.com/regulatory/hog-producers-negatively-impacted-usda-decision.

[CIT0002] AOAC. 2005. Official methods of analysis of AOAC International. Gaithersburg (MD): AOAC International.

[CIT0003] Bruininx, E. M., G. P. Binnendijk, C. M. van der Peet-Schwering, J. W. Schrama, L. A. den Hartog, H. Everts, and A. C. Beynen. 2002. Effect of creep feed consumption on individual feed intake characteristics and performance of group-housed weanling pigs. J. Anim. Sci. 80:1413–1418. doi:10.2527/2002.8061413x. 12078720

[CIT0004] Cera, K. R., D. C. Mahan, R. F. Cross, G. A. Reinhart, and R. E. Whitmoyer. 1988. Effect of age, weaning and postweaning diet on small intestinal growth and jejunal morphology in young swine. J. Anim. Sci. 66:574–584. doi:10.2527/jas1988.662574x. 3372395

[CIT0002a] Fuller, M. 2012. Determination of protein and amino acid digestibility in foods including implications of gut microbial amino acid synthesis. Br. J. Nutr. 108:238–246. doi:10.1017/s0007114512002279. 23107534

[CIT0005] Huber, L. A., S. Hooda, R. E. Fisher-Heffernan, N. A. Karrow, and C. F. M. de Lange. 2018. Effect of reducing the ratio of omega-6-to-omega-3 fatty acids in diets of low protein quality on nursery pig growth performance and immune response. J. Anim. Sci. 96:4348–4359. doi:10.1093/jas/sky296. 30053222 PMC6162592

[CIT0006] Kim, J. H., K. N. Heo, J. Odle, K. Han, and R. J. Harrell. 2001. Liquid diets accelerate the growth of early-weaned pigs and the effects are maintained to market weight. J. Anim. Sci. 79:427–434. doi:10.2527/2001.792427x. 11219452

[CIT0007] Koo, B., J. Choi, C. Yang, and C. M. Nyachoti. 2020. Diet complexity and l-threonine supplementation: effects on growth performance, immune response, intestinal barrier function, and microbial metabolites in nursery pigs. J. Anim. Sci. 98:1–11. doi:10.1093/jas/skaa125. PMC7229884 32307528

[CIT0008] Lafleur Larivière, E., C. Zhu, S. Zettell, R. Patterson, N. A. Karrow, and L. Huber. (2021). The effect of deoxynivalenol-contaminated corn and an immune-modulating feed additive on growth performance and immune response of nursery pigs fed corn- and soybean meal-based diets. Transl. Anim. Sci. 5. doi:10.1093/tas/txab141. PMC8485908 34611597

[CIT0009] Miller, Y. J., A. M. Collins, R. J. Smits, P. C. Thomson, and P. K. Holyoake. 2012. Providing supplemental milk to piglets preweaning improves the growth but not survival of gilt progeny compared with sow progeny. J. Anim. Sci. 90:5078–5085. doi:10.2527/jas.2011-4272. 22829606

[CIT0010] Muns, R., and E. Magowan. 2018. The effect of creep feed intake and starter diet allowance on piglets’ gut structure and growth performance after weaning. J. Anim. Sci. 96:3815–3823. doi:10.1093/jas/sky239. 29924319 PMC6127789

[CIT0025] Myers, W. D., P. A. Ludden, V. Nayigihugu, and B. W. Hess. 2004. A procedure for the preparation and quantitative analysis of samples for titanium dioxide. J. Anim. Sci. 82:179–183. doi:10.2527/2004.821179x. 14753360

[CIT0011] Novotni-Dankó, G., P. Balogh, L. Huzsvai, and Z. Gyori. 2015. Effect of feeding liquid milk supplement on litter performances and on sow back-fat thickness change during the suckling period. Arch. Anim. Breed. 58:229–235. doi:10.5194/aab-58-229-2015.

[CIT0012] NRC. 2012. Nutrient requirements of swine. 11th ed. Washington (DC): National Academy Press.

[CIT0026] Nyachoti, C. M., C. F. M. de Lange, B. W. McBride, S. Leeson, and H. Schulze. 2000. Dietary influence on organ size and in vitro oxygen consumption by visceral organs of growing pigs. Livest. Prod. Sci. 65:229–237. doi:10.1016/S0301-6226(00)00157-3.

[CIT0013] van Oostrum, M., A. Lammers, and F. Molist. 2016. Providing artificial milk before and after weaning improves postweaning piglet performance. J. Anim. Sci. 94:429–432. doi:10.2527/jas2015-9732.

[CIT0014] Pajor, E. A., D. Fraser, and D. L. Kramer. 1991. Consumption of solid food by suckling pigs: individual variation and relation to weight gain. Appl. Anim. Behav. Sci. 32:139–155. doi:10.1016/S0168-1591(05)80038-3.

[CIT0027] Park, B. C., D. M. Ha, M. J. Park, and C. Y. Lee. 2014. Effects of milk replacer and starter diet provided as creep feed for suckling pigs on pre- and post-weaning growth. Anim. Sci. J. 85:872–878. doi:10.1111/asj.12246. 25039284

[CIT0015] Piñeiro, C., A. Manso, E. G. Manzanilla, and J. Morales. 2019. Influence of sows’ parity on performance and humoral immune response of the offspring. Porcine Health Manag. 5:1. doi:10.1186/s40813-018-0111-8. 30783536 PMC6375126

[CIT0016] Pluske, J. R., D. J. Kerton, P. D. Cranwell, R. G. Campbell, B. P. Mullan, R. H. King, G. N. Power, S. G. Pierzynowski, B. Westrom, C. Rippe, et al. 2003. Age, sex, and weight at weaning influence organ weight and gastrointestinal development of weanling pigs. Aust. J. Agric. Res. 54:515–527. doi:10.1071/AR02156.

[CIT0017] Pluske, J. R., J. C. Kim, C. F. Hansen, B. P. Mullan, H. G. Payne, D. J. Hampson, J. Callesen, and R. H. Wilson. 2007. Piglet growth before and after weaning in relation to a qualitative estimate of solid (creep) feed intake during lactation: a pilot study. Arch. Anim. Nutr. 61:469–480. doi:10.1080/17450390701664249. 18069618

[CIT0018] Skinner, L. D., C. L. Levesque, D. Wey, M. Rudar, J. Zhu, S. Hooda, and C. F. de Lange. 2014. Impact of nursery feeding program on subsequent growth performance, carcass quality, meat quality, and physical and chemical body composition of growing-finishing pigs. J. Anim. Sci. 92:1044–1054. doi:10.2527/jas.2013-6743. 24492546

[CIT0019] Sulabo, R. C., M. D. Tokach, J. M. Derouchey, S. S. Dritz, R. D. Goodband, and J. L. Nelssen. 2010. Influence of feed flavors and nursery diet complexity on preweaning and nursery pig performance. J. Anim. Sci. 88:3918–3926. doi:10.2527/jas.2009-2724. 20833770

[CIT0020] Wolter, B. F., M. Ellis, B. P. Corrigan, and J. M. DeDecker. 2002. The effect of birth weight and feeding of supplemental milk replacer to piglets during lactation on preweaning and postweaning growth performance and carcass characteristics. J. Anim. Sci. 80:301–308. doi:10.2527/2002.802301x. 11881919

[CIT0021] Zijlstra, R. T., K. Y. Whang, R. A. Easter, and J. Odle. 1996. Effect of feeding a milk replacer to early-weaned pigs on growth, body composition, and small intestinal morphology, compared with suckled littermates. J. Anim. Sci. 74:2948–2959. doi:10.2527/1996.74122948x. 8994909

